# Not too close! impact of roommate status on MRSA and VRE colonization and contamination in Nursing Homes

**DOI:** 10.1186/s13756-021-00972-1

**Published:** 2021-07-05

**Authors:** Marco Cassone, Meghan Linder, Cheon Jee Shin, Julia Mantey, Kristen Gibson, Bonnie Lansing, Lona Mody

**Affiliations:** 1 Division of Geriatric and Palliative Care Medicine, Department of Internal Medicine, University of Michigan Medical School, Ann Arbor, MI 3023 BSRB, 109 Zina Pitcher Place48109 USA; 2 School of Public Health, University of Michigan, Ann Arbor, MI USA; 3 Geriatric Research and Education Clinical Center, VA Ann Arbor, Ann Arbor, MI USA; 4 Present Address: Oregon Health Authority, Portland, OR USA; 5 Present Address: University of Louisville, Louisville, KY USA

**Keywords:** Multiple occupancy, Antimicrobial resistance, Nursing Homes, VRE, *S. aureus*, Environment, Double room, Colonization, Contamination, Infection prevention

## Abstract

Multiple room occupancy is common in Nursing Homes (NHs), and its role in transmission of antibiotic-resistant pathogens is unclear. We investigated prevalence of patient colonization and environmental contamination with vancomycin-resistant enterococci (VRE) and methicillin-resistant *Staphylococcus aureus* (MRSA) in NH roommates, compared it with expected prevalence, and determined specific body and environmental sites that may act as sources of roommate colonization. Roommate contamination was associated with index patient’s colonization (relative risk (RR): 2.57 (95% CI 1.04–6.37)) for MRSA, and index patient’s immediate environment contamination for VRE (RR: 3.60 (95% CI 1.59–8.12)). When specific index patient sites associated with roommate colonization were investigated, the side table (Fisher’s p = 0.029 and 0.047 for VRE and MRSA, respectively) and the nurse call button (p = 0.001 and 0.052) stood out, together with patient hands in the case of VRE (p = 0.026). Future studies should be carried out to establish whether these sites should be a specific target of infection prevention campaigns in NHs with multiple occupancy rooms.

**Supplementary Information:**

The online version contains supplementary material available at 10.1186/s13756-021-00972-1.

Patient colonization and environmental contamination with multidrug-resistant organisms are common in Nursing Homes (NHs), where multiple room occupancy is a popular option. Vancomycin-resistant enterococci (VRE) and methicillin-resistant *Staphylococcus aureus* (MRSA) are among the most commonly found antimicrobial-resistant pathogens in this vulnerable population, where they are found as often or more often than in acute care settings [1–3]. Prior studies have shown that VRE and MRSA shedding and cross-transmission occur often and may be associated with unfavorable health outcomes [1,4–6].

In one intensive care unit study, it has been demonstrated that patients can be significantly more likely to acquire VRE and MRSA if the prior room occupant was colonized with the same organism 7. Data is scarce for non-critical care and for the expanding long-term health care setting, with inconsistent results reported for VRE, and no significant risk of MRSA colonization 8. Additionally, the role and contribution of environmental contamination has often been overlooked.

Globally, caring for older adults in shared spaces is common. Since sharing rooms can lead to transmission of pathogens, we aimedto assess whether co-colonization and co-contamination with MRSA and VRE occurs more often than expected among roommates, to investigate the probability of colonization and contamination in roommates of a colonized patient, and to determine if and which specific source sites may be driving such burden.

## Methods

### Patient population and sampling

The present study is based on an in depth analysis of a subset of patients enrolled in a larger prospective cohort study of newly admitted patients in southeast Michigan NHs [9]. Patient pair visits were eligible to be included in the present study if they were both enrolled in the parent study, and sharing a double occupancy room at the time of the visit. The patient who was visited at an earlier date was noted as “index patient”, while the second one was noted as “roommate”. In cases where sampling was performed on the same day for both patients, the patient who had been admitted to the room earlier was noted as the “index patient”. Four or more body sites (hands, nares, oral cavity, groin, and perianal area), and four or more environmental sites in the proximity of each patient (bedrail, bed controls, nurse call button, side table top and bottom, and TV remote control) were sampled. Contamination of shared environmental sites (i.e.: sites that could have been touched by both patients, such as the toilet seat and the privacy curtains) was not considered for this study. For each of the two pathogens of interest, patient body colonization and environmental contamination were considered to be present based on the positivity of one or more body or environmental sites, respectively.

### Laboratory methods

Samples were collected using Bactiswabs (Remel, Lenexa, Kansas), and processed on the same day. Nares, oral, groin and perianal samples were streaked on TSA 5% sheep blood agar plates, MSA agar, and bile-esculin agar with 6 ug/ml vancomycin (BEV6) and incubated for up to 48 h at 36 °C. All other samples were enriched overnight in BHI broth, after which they were streaked and incubated as above. Growth suggestive of staphylococci on MSA was tested by catalase and coagulase test (Staphaurex, Remel, Lenexa, KS), and *S. aureus* isolates were screened for methicillin resistance using cefoxitin disc diffusion according to CLSI criteria. Growth suggestive of VRE on BEV6 was confirmed by pyrrolidonyl arylamidase testing (DrySlide, BD, Franklin Lakes, NJ).

### Statistical analysis

Colonization and contamination with MRSA and VRE were analyzed independently. Colonization and contamination rates were obtained for all enrolled patients and their immediate environment, respectively, at their first visit. To determine whether double room placement may result in increased likelihood of colonization and/or contamination, we compared actual frequencies of co-colonization in roommates to frequencies expected in the overall population of tested patients (i.e.: the square of all tested patients’ colonization rates), using a simple proportion comparison test (two-tailed *z* test). The overall tested patient population included patients with roommates of unknown status and patients with no roommate.

We then considered each visit when microbiological data was available for both occupants of double rooms: in this case we calculated the relative risk (RR) of colonization of a roommate when the index patient sharing her/his room was colonized compared with when the index patient was not colonized. Similarly, we calculated the RR for environmental contamination of the roommate surrounding based on index patient’s surrounding contamination. and for the cross probabilities of body colonization and environmental contamination.

Finally, to establish if and which body and/or environmental site/s may be specifically associated with an outsized risk of roommate colonization or contamination, we employed a site-specific strategy, by comparing rates for each sampling site in index patients and their immediate vicinity, according to their roommate’s overall colonization or contamination status (Fisher’s exact test).

## Results

### Expected and observed prevalence of MRSA and VRE colonization of both roommates

Microbiological data was available on body and environmental MRSA and VRE burden for 1619 visits of 651 patients. In 56 cases, microbiological data was available for both patients sharing a double room. The rate of MRSA body colonization and room contamination upon admission for all patients with available microbiological data, and regardless of room occupancy status (single or double) was 0.161 (16.1%) and 0.171 (17.1%), respectively. Thus, the prevalence of dual colonization and contamination of both roommates, upon the assumption that their statuses are independent, would be expected to be 0.161^2^ (2.6%) and 0.171^2^ (2.9%). Actual observations revealed 7.1% for both colonization (p = 0.028) and contamination (p = 0.054), pointing to a possible interdependency of MRSA status between roommates. For VRE, the prevalence of body colonization and room contamination among all patients was 33.2% in both cases, leading to an expected dual percentage of 11.0%. Actual data showed 21.4% contamination (p = 0.071), and 8.9% colonization. A relatively high prevalence of VRE was expected in such NH patients [6].

### Risk of colonization and contamination in roommates of colonized patients

In visits where matched microbiological data was available for both patients, index patient MRSA colonization translated to a higher risk of contamination of their roommate’s immediate environment (RR 2.57, 95% CI 1.04–6.37, p = 0.04), and a higher risk of roommate colonization, although not reaching statistical significance (RR 2.62 (0.74–9.26), p = 0.08) (Table 1). Similar results were obtained when the opposite scenario was investigated, namely the likelihood of roommate body colonization based on the index patients’ immediate environment status (RR 3.31 (0.96–11.4), p = 0.05). Conversely, no evidence was found of an association between index patient and roommate environmental contamination (RR 1.65 (0.59–4.62), p = 0.35).

**Table 1:** Roommate colonization and contamination in patients and their surroundings as a function of index patient status

Pathogen	Index patient status	Roommate outcome	Roommate positivity rates. N. positives/total (%)		
			Negative index patient	Positive index patient	Relative Risk (95% CI)
MRSA	Colonization	Colonization	4/42 (9.5%)	4/14 (28.6%)	2.62 (0.74–9.26)
	Colonization	Contamination	7/42 (16.7%)	6/14 (42.9%)	**2.57 (1.04–6.37)**
	Contamination	Contamination	8/43 (18.6%)	4/13 (30.8%)	1.65 (0.59–4.62)
	Contamination	Colonization	4/43 (9.3%)	4/13 (30.8%)	3.31 (0.96–11.4)
VRE	Colonization	Colonization	15/41 (36.6%)	5/15 (33.3%)	0.91 (0.40–2.07)
	Colonization	Contamination	8/40 (20%)	6/16 (37.5%)	1.87 (0.77–4.54)
	Contamination	Contamination	6/36 (16.7%)	12/20 (60.0%)	**3.60 (1.59–8.12)**
	Contamination	Colonization	9/35 (25.7%)	9/21 (42.9%)	1.67 (0.79–3.52)

Relative Risk values in bold represent statistical significance (p < 0.05)

The opposite was found for VRE: in this case, there was a strong relationship between environmental contamination of index patient’s and roommate’s immediate surroundings (RR 3.60 (1.59–8.12), p = 0.002) compared to a weaker evidence of cross contamination between the index patient and their roommate’s environment, (RR 1.87 (0.77–4.54), p = 0.17 and 1.67 (0.79–3.52), p = 0.18, respectively).

### Potential source sites of roommate MRSA and VRE acquisitions

**Fig. 1:**
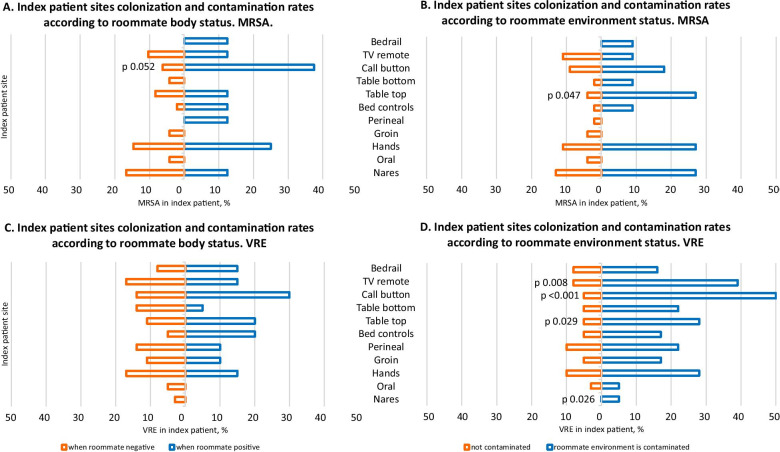
Rates of colonization/contamination of specific body/environmental sites among index patients of colonized and non-colonized roommates. **A** MRSA rates according to roommate colonization. **B** MRSA rates according to roommate immediate environment contamination. **C** VRE rates according to roommate colonization. **D** VRE rates according to roommate immediate environment contamination

Among specific sampling sites, the nurse call button appears to be more likely to be contaminated in index patients of MRSA-colonized roommates (p = 0.052), and the side table (top) in index patients of roommates with MRSA-contaminated surroundings (p = 0.047) (Fig. 1). In the case of VRE, multiple index patient environmental sites appear to be significantly more often contaminated with VRE when their roommates’ environment is also contaminated (nurse call button, TV remote control, and side table top, p values ranging from < 0.001 to 0.029) (Fig. 1).

Among index patient body sites, the hands are the most strongly associated with roommate burden, including a statistically significant association when roommates’ immediate environment is contaminated with VRE (p = 0.026). Taken together, these observations indicate that not only patient colonization, but also environmental contamination may be associated with higher MRSA and VRE burden amongst patients sharing a room.

## Discussion and conclusions

Our original data suggests that MRSA and VRE colonization of a NH patient residing in a double room may be a risk factor for acquisition by her/his roommate, and points to patient hands as well as certain environmental sites as possible sources for transmission 1. We now show a higher rate of MRSA colonization in roommates of colonized patients in NHs, and also report a similar association for VRE among roommates’ immediate surroundings. Reports in the acute care literature have shown a potential role of room sharing in the build-up to epidemics, especially for beta-lactamases- and carbapenemase-carrying gram-negatives 10,11, and implied a probable role of the environment in transmission of MRSA and VRE between consecutive patients in intensive care unit rooms 7]. A recent meta-analysis of literature reported contrasting findings for VRE and no clear association for MRSA in non-acute care patients [8, including in NH settings 12. Studies in endemic as opposed to outbreak settings, especially outside of acute-care, are urgently needed in order to establish roommate risk in NHs, an environment in which double and multiple occupancy is still very common and where length of stay is often measured in weeks rather than days, leading to potentially more opportunities for in-room transmission. In addition, our present findings underline the role that patient hands may play in the transmission of pathogens in healthcare settings. Unlike the case of healthcare workers’ hands, such a role has scarcely been investigated before [13].

Interestingly, MRSA has been demonstrated to be capable of colonizing up to 12% of roommates of positive patients in double occupancy hospital rooms 14. Regarding VRE, studies in intensive care units have demonstrated that room contamination is a risk factor for patient acquisition 15,16, and that contamination rates in the room can be as high as 10% even after terminal cleaning. However, such environmental burden does not necessarily result in higher colonization risk for roommates or for patients later admitted to the room 16. Our observations, including the association with patient hands and environmental burden, suggests that additional studies from geographically diverse communities should be conducted.

This study was not powered to prove transmission and its direction, thus the implication of index patient as a “source” must be taken with caution in the face of limited understanding of NH colonization and contamination dynamics. Still, it must be noted that the sub-population of patients from which our roommate pair visit data originated was not different from the general study population in terms of potential confounding risk factors such as age, sex, comorbidity and functional status (Additional file 1: Table S1). Nevertheless, the need for further, in depth research on pathogens’ transmission pathways in NHs is readily apparent as our understanding is just starting to coalesce into some basic guiding principles. The resulting knowledge would be critical to establish whether additional surveillance screening (including environmental screening), improved cleaning, and patient-targeted education should be implemented for patients placed in multiple occupancy rooms, even as we consider whether the multiple occupancy model should be gradually phased out.

## Supplementary Information

**Additional file 1.** Antimicrobial usage for each facility.

## Data Availability

The datasets used during the current study are available from the corresponding author on reasonable request.

## Abbreviations

NH: Nursing Home
MRSA: Methicillin-resistant *Staphylococcus aureus*
VRE: Vancomycin-resistant *Enterococcus*
MSA: Mannitol salt agar
BEV6: Bile-esculin agar with 6 ug/ml vancomycin
RR: Relative risk
