# Supplementary material for: Not too close! impact of roommate status on MRSA and VRE colonization and contamination in Nursing Homes
Source: Antimicrob Resist Infect Control. 2021 Jul 5;10:104. doi: 10.1186/s13756-021-00972-1 (PMC8258944; doi:10.1186/s13756-021-00972-1)
Supplement: Supplementary file 1 — Additional file 1. Antimicrobial usage for each facility. [file 13756_2021_972_MOESM1_ESM.docx]

Supplemental Table.

Antimicrobial usage for each Facility: number of patients receiving antibiotics at any time during their stay in the facility. Cross-sectional data obtained from facility records.

| Facility |  | 1 | 2 | 3 | 4 | 5 | 6 |  | Total |
| --- | --- | --- | --- | --- | --- | --- | --- | --- | --- |
|  |  |  |  |  |  |  |  |  |  |
| N. patients receiving antibiotics |  | 97  (73%) | 42  (52%) | 118  (71%) | 96  (70%) | 36  (68%) | 48  (65%) |  | 437  (68%) |
| Total N. patients |  | 133 | 80 | 167 | 137 | 53 | 74 |  | 644* |

* Data not available for 7 patients
